# Child health inequities in developing countries: differences across urban and rural areas

**DOI:** 10.1186/1475-9276-5-9

**Published:** 2006-07-11

**Authors:** Jean-Christophe Fotso

**Affiliations:** 1 African Population & Health Research Center (APHRC), P.O. Box 10787, 00100 GPO, Nairobi, Kenya

## Abstract

**Objectives:**

To document and compare the magnitude of inequities in child malnutrition across urban and rural areas, and to investigate the extent to which within-urban disparities in child malnutrition are accounted for by the characteristics of communities, households and individuals.

**Methods:**

The most recent data sets available from the Demographic and Health Surveys (DHS) of 15 countries in sub-Saharan Africa (SSA) are used. The selection criteria were set to ensure that the number of countries, their geographical spread across Western/Central and Eastern/Southern Africa, and their socioeconomic diversities, constitute a good yardstick for the region and allow us to draw some generalizations. A household wealth index is constructed in each country and area (urban, rural), and the odds ratio between its uppermost and lowermost category, derived from multilevel logistic models, is used as a measure of socioeconomic inequalities. Control variables include mother's and father's education, community socioeconomic status (SES) designed to represent the broad socio-economic ecology of the neighborhoods in which families live, and relevant mother- and child-level covariates.

**Results:**

Across countries in SSA, though socioeconomic inequalities in stunting do exist in both urban and rural areas, they are significantly larger in urban areas. Intra-urban differences in child malnutrition are larger than overall urban-rural differentials in child malnutrition, and there seem to be no visible relationships between within-urban inequities in child health on the one hand, and urban population growth, urban malnutrition, or overall rural-urban differentials in malnutrition, on the other. Finally, maternal and father's education, community SES and other measurable covariates at the mother and child levels only explain a slight part of the within-urban differences in child malnutrition.

**Conclusion:**

The urban advantage in health masks enormous disparities between the poor and the non-poor in urban areas of SSA. Specific policies geared at preferentially improving the health and nutrition of the urban poor should be implemented, so that while targeting the best attainable average level of health, reducing gaps between population groups is also on target. To successfully monitor the gaps between urban poor and non-poor, existing data collection programs such as the DHS and other nationally representative surveys should be re-designed to capture the changing patterns of the spatial distribution of population.

## 1. Background

African cities have experienced tremendous population growth over the last few decades, and most of the future population growth in the region is expected to occur in urban areas [1]. Unfortunately, this rapid pace of urbanization has been occurring amidst declining economies, leading to inability of local and national authorities to provide basic social services and employment opportunities to the growing urban population [2]. Recent estimates show that urban population in sub-Saharan Africa (SSA) grew by almost 4.7% per year between 1980 and 2000 [1], while per capita gross domestic product (GDP) dropped annually by nearly 0.8% [3]. It is generally admitted that the impact of economic restructuring since the 1980s has been most severe on residents of major cities in SSA, following reduced public expenditure on municipal services, housing and infrastructure [4]. Consequently, urban population explosion in developing countries and in SSA in particular, is accompanied by increasing urban poverty and malnutrition [2,5].

Newly assembled evidence from developing countries indicates that the locus of poverty and malnourishment is gradually shifting from rural to urban areas, as the number of urban poor and undernourished is increasing more quickly than the rural number [6]. This trend is also illustrated by the narrowing urban-rural gap in child malnutrition in most countries of SSA [7]. One of the distinct faces of urban poverty in SSA is the proliferation of overcrowded slums and shantytowns characterized by unhygienic environmental conditions (e.g. uncollected garbage, unsafe water, poor drainage and open sewers) which worsen the susceptibility of residents to various health problems [2,8]. As a result of such unhealthy conditions, rates of child malnutrition, morbidity and mortality are several times higher in slums and peri-urban areas than in more privileged urban neighborhoods, and even than in rural areas [4,9].

The evidence of large and even widening inequalities in health between the rich and the poor has stimulated international and national organizations to focus explicitly on the health and nutrition of the poor in the developing world [10-12]. The focus on the poor is premised on the reality that the resulting poor health hinders human capital, thereby creating and perpetuating a vicious circle of poverty and poor health [6,13]. Thus, addressing the problems of inequalities in child health, both between countries and within countries, remains one of the greatest challenges, especially for policies and programs related to the Millennium Developments Goals (MDG) [10]. The World Health Organization (WHO) corroborated the focus on improving the health of the most vulnerable and reducing inequalities between population subgroups and stated that *"the objective of good health is twofold: the best attainable average level, and the smallest feasible differences among individuals" *[14].

Against this background, the purpose of this paper is to contribute to the growing empirical literature on socioeconomic inequalities in health in developing countries, by examining differences across urban and rural areas in health inequalities. Specifically, the goals of this study are: (1) to document and compare the magnitude of inequities in child malnutrition across urban and rural areas; and (2) to investigate the extent to which socioeconomic inequalities^1 ^in urban areas are accounted for by the characteristics of communities, households and individuals. Given that urbanization has been one of the dominant underlying demographic processes in the past few decades not only in SSA, but also in the rest of the developing world, one of the key concerns is the extent of socioeconomic disparities in child health across urban and rural areas. Indeed, health-related resource allocation decisions generally rely on simple urban-rural comparisons, which mask the enormous disparities that are increasingly evidenced between socioeconomic subgroups in urban areas [5].

The focus on malnutrition among children is predicated on the fact that undernutrition is one of the major public health concerns in developing countries, where it represents both a cause and a manifestation of poverty [13,15,16]. The evidence of short and long-term consequences of nutritional deficiencies include increased risk of both morbidity from infectious diseases and mortality, impaired cognitive or delayed mental development and, subsequently, reduced learning abilities in school, and poor work capacity in adulthood [17,18]. Conversely, child undernutrition in developing countries is usually a consequence of poverty, with its attributes of low family income, poor education, poor environment and housing, and inadequate access to foods, safe water and health care services [16,19]. Investigating socioeconomic inequalities in child malnutrition within SSA is of special importance since the region is not on target to reach the MDGs. Recent data indicate that whereas malnutrition among preschoolers is substantially decreasing in Asia and Latin America and the Caribbean, it is on the rise in some countries of SSA, whilst in many others they remain disturbingly high or are declining only sluggishly [17].

## 2. Data and methods

### 2.1. Data and selected countries

This research uses the most recent data sets available as of January 2005 from the Demographic and Health Surveys (DHS) of the following 15 countries: Burkina Faso, Cameroon, Chad, Côte d'Ivoire, Ghana, Nigeria, and Togo from Western and Central Africa, and Kenya, Madagascar, Malawi, Mozambique, Tanzania, Uganda, Zambia and Zimbabwe from Eastern and Southern Africa. The selection criteria were not only based on the availability of data on child nutritional status, but more importantly, were set to ensure that the number of selected countries, their geographical spread across Western/Central and Eastern/Southern Africa, and their socioeconomic diversities, could allow us to draw some generalizations. Indeed, Column (Col.) 1 of Table 1 shows that according to the human development index (HDI^2^), four countries (Ghana, Zimbabwe, Cameroon and Kenya) can be classified as high-HDI (ranking below 20 out of 48 African countries); six others (Madagascar, Togo, Nigeria, Zambia, Côte d'Ivoire and Tanzania) are middle-HDI (ranking between 20 and 30); and the five remaining (Burkina Faso, Mozambique, Chad, Malawi and Uganda) can be classified as low-HDI (ranking 31 and higher). Further, in each of the above categories of ranking, there is almost the same number of countries from either region (Central/Western and Eastern/Southern Africa).

**Table 1:** Human development index, urban population and gross domestic product in 15 selected countries

	Human Development Index (HDI) ranking^a^	Percentage of urban population^b^	Urban population annual growth rate^b^	Gross domestic product per capita^c^	
				
				Value	Annual variation (%)
			
	2000 (1)	2000 (2)	198s0–2000 (3)	2000 (4)	1980–2000 (5)
Central & Western Africa					
1. Burkina Faso	46	16.7	6.4	270	1.2
2. Cameroon	16	49.0	5.1	664	-0.4
3. Chad	41	23.8	4.0	205	0.7
4. Côte d'Ivoire	28	43.6	4.4	821	-1.7
5. Ghana	12	43.9	4.7	407	0.3
6. Nigeria	25	44.1	5.5	255	-1.0
7. Togo	22	33.4	5.0	320	-1.9
Eastern & Southern Africa					
8. Kenya	18	35.9	7.4	328	-0.1
9. Madagascar	20	26.0	4.6	246	-1.7
10. Malawi	37	15.1	5.7	168	0.2
11. Mozambique	42	32.1	6.6	191	0.9
12. Tanzania	30	32.3	7.2	192	0.5
13. Uganda	32	12.0	4.8	339	2.1
14. Zambia	27	35.1	2.2	404	-1.8
15. Zimbabwe	13	33.6	5.0	619	0.1
					
All 15 countries	NAp^d^	35.6	5.4	323	-0.7
Sub-Saharan Africa	NAp	34.0	4.7	572	-0.8
Developing countries	NAp	40.5	3.5	NAv^e^	NAv

^a^Ranking within 48 African countries. Countries are ranked in decreasing order of human development index. Source: United Nations Development Program, 2000.

^b^Source: United Nations, 2004.

^c^At constant 1995 US$. Available data for Uganda and Tanzania start in 1982 and 1988 respectively. Source: World Bank, 2004.

^d^NAp: Not applicable;^e^NAv: Not available.

Table 1 also illustrates the economic diversity of the selected countries with regard to levels of urbanization and per capita gross domestic product (GDP) in 2000. It shows that the percentage of urban population (Col. 2) differs significantly among the selected countries. It varies from 12–17% in Uganda, Malawi and Burkina Faso, to close to or more than 45% in Cameroon, Nigeria, Ghana and Côte d'Ivoire. The average value for SSA is 34%. As for GDP per capita, Côte d'Ivoire, Cameroon and Zimbabwe emerge as the most affluent countries with values higher than $600, whilst by contrast Malawi, Mozambique, Tanzania, Chad and Madagascar are the most deprived (less than $250). The selected countries also display marked socioeconomic diversities in terms of per capita food production, per capita health expenditures, and adult literacy rates (not shown). Overall, we make no pretence that the sample countries are representative of the entire SSA, but their number and geographical and socioeconomic diversities constitute a good yardstick for the region and help to strengthen the findings from the study.

Moreover, the selected countries typify rapid urbanization amidst declining economies. Table 1 shows that between 1980 and 2000, the urban population grew by 5.4% per year in the selected countries as a whole, against an average of 3.5% for developing countries. The fastest growths are recorded in Kenya (7.4%), Tanzania (7.2%) and Mozambique (6.6%). By contrast, Zambia (2.2%), Chad (4.0%) and Côte d'Ivoire (4.4%) witnessed the slowest growth rates of their urban populations. At the same time, GDP per capita dropped by 0.7% on average in the selected countries. The most marked reductions are in Togo, Zambia, Cote d'Ivoire and Madagascar (1.7–1.9%), whereas improvements are recorded in Uganda (+2.1%) and Burkina Faso (1.2%), and to a lesser degree in Mozambique (0.9%) and Chad (0.7%).

### 2.2. Dependent variable

Among various growth-monitoring indices, the three most commonly used profiles of malnutrition in children are stunting, wasting and underweight, measured by height-for-age, weight-for height, and weight-for-age indexes, respectively. The present study focuses on stunting (or growth retardation) in young children. Stunting results from recurrent episodes or prolonged periods of nutrition deficiency for calories and/or protein available to the body tissues, inadequate intake of food over a long period of time, or persistent or recurrent ill-health [15,18]. Since the height-for-age measure is less sensitive to temporary food shortages, stunting is considered the most reliable indicator of a child's nutritional status, especially for the purpose of differentiating socioeconomic conditions within and between countries [20,21]. As recommended by the WHO, children whose indices fall more than two standard deviations below the median of the NCHS/CDC/WHO reference population are classified as stunted [17].

### 2.3. Measuring socioeconomic inequalities in child health

Despite the growing number of studies attesting evidence of poorer health among people with less education and income, lower status jobs, and poorer housing [12,21-25], there is still debate about the meaning of health inequalities [26-28]. Kawachi *et al*. arguably state that priority must be given to analysing health inequalities between groups, referred to as health inequities [29]. There is also a great deal of discussion on the appropriate measures to capture such inequities [30,31]. The concentration index is increasingly used in the literature on socioeconomic inequalities in health [12,21,22,25]. The concentration curve plots the cumulative proportions of the population (beginning with the most disadvantaged) against the cumulative proportion of the health outcome under study. The resulting concentration index which varies from -1 to +1 measures the extent to which a health outcome is unequally distributed across groups [25]. Though this measure takes into account what is going on in all the groups, it is mainly used for descriptive purposes, and adjustment for control variables is not straightforward. The odds ratio between the uppermost and the lowermost categories of the socioeconomic variable is used in this paper as a proxy for socioeconomic inequalities. The main advantage of this approach is the use of a single number which makes it easier to compare the magnitude of inequalities across populations or over time, even though it overlooks the health outcome in the intermediate groups of the socioeconomic variable. This measure is particularly appropriate when a linear trend has previously been observed in the association between the socioeconomic variable and the health outcome under consideration [30].

Poverty -and thus SES- has been recognized to be multi-faceted, and to exert its influences on health at various levels (individual, household, community and nation). Poverty includes, but is not limited to, inadequate income, shelter and assets for individuals and households, and inadequate provision of infrastructure and basic services such as health services, roads, schools and vocational training [19,32]. This paper privileges the economic and material dimension of poverty at the household level. DHS data do not provide information on income or expenditures. Thus, along the lines of Gwatkin *et al*. and Filmer and Pritchett [33,34], we build on our previous work [35] and construct a household wealth index in each country and area (urban, rural). The wealth index is constructed from household's possessions, source of drinking water, type of toilet facilities and flooring material using principal components analysis. It is then re-coded as poorest (bottom 30%), middle (next 40%), and richest (top 30%), with poorest as the reference category.

### 2.4. Control variables

The key control variables used in the study include urban-rural place of residence, and maternal education, known to have some effects on child health and nutrition that are independent of the effects of other measures of SES [23,36]. Maternal education is coded as no education (reference category), primary, secondary or higher. The controls also include a community SES constructed in each country and area, from the proportion of households having access to clean water and electricity, as well as the proportion of wage earners and that of educated adults (level of primary education or higher). The variable, which is in line with the multilevel nature of the health determinants [16,37-39], is designed to represent the broad socio-economic ecology of the neighborhoods in which families live, besides the broad rural-urban location of residence. Father's education is also used in this study. In some societies of the developing world, certain behaviors and practices which may affect child health and nutrition are highly dependent on characteristics of the father, particularly his level of education [22]. The other control variables used in this study include: (i) at the mother level: age at birth of the index child, marital status, religion, and nutritional status; and (ii) at the child level: current age, sex, low birth weight, antenatal care, place of delivery, age-specific immunization status, birth order and interval, and breast feeding duration.

### 2.5. Statistical methods

DHS data have a hierarchical structure, with children nested within mothers, mothers clustered within households, and households nested within communities. As a result, observations from the same group are expected to be more alike at least in part because they share a common set of characteristics or have been exposed to a common set of conditions, thus violating the standard assumption of independence of observations inherent in conventional regression models. Consequently, unless some allowance for clustering is made, standard statistical methods for analyzing such data are no longer valid, as they generally produce downwardly biased variance estimates, leading for example to infer the existence of an effect when, in fact, that effect estimated from the sample could be ascribed to chance [40,41]. Multilevel models provide a framework for analysis which is not only technically stronger, but which also has a much greater capacity for generality than traditional single-level statistical methods [42]. Given that the number of children per household in the data for this analysis is very small (between 1.1 and 1.3), we carry out two-level (child and community) logistic regression analyses in each country and area. Models are fitted using the MLwiN software with Binomial, Predictive Quasi Likelihood (PQL) and second-order linearization procedures [41].

## 3. Results

### 3.1. Descriptive analyses

The selected countries, years of data collection and sample sizes are shown in Table 2. Only children under three years of age were included in the samples to ensure strict comparability across countries. Further, children with missing or inconsistent anthropometric measures were excluded from the sample. The percentage of omission due to missing or inconsistent anthropometric measurements varied from 6–10% in Zambia, Tanzania, Kenya, Malawi, Ghana and Côte d'Ivoire to 15%-20% in Cameroon, Zimbabwe, Mozambique and Burkina Faso.

**Table 2:** Sample characteristics

	Survey year	Number of children^a^	Percentage of urban children	Percentage of stunted children			Rural to urban odds ratio
					
				Overall	Urban	Rural	
Central & Western Africa							
1. Burkina Faso	1998/99	2 428	12.0	31.4	20.6	32.9	1.9
2. Cameroon	1998	1 763	26.5	30.2	22.9	32.8	1.6
3. Chad	1996/97	3 416	21.2	35.9	28.3	37.9	1.5
4. Côte d'Ivoire	1998/99	986	33.3	22.5	18.0	24.8	1.5
5. Ghana	2003	1 894	33.1	27.3	20.0	30.9	1.8
6. Nigeria	2003	2 713	32.3	36.5	29.2	40.0	1.6
7. Togo	1998	3 399	23.6	22.3	15.2	24.5	1.8
Eastern & Southern Africa							
8. Kenya	2003	2 912	17.9	30.7	24.3	32.0	1.5
9. Madagascar	1997	2 879	19.5	49.0	45.3	50.0	1.2
10. Malawi	2000	5 936	13.2	44.6	29.7	46.9	2.1
11. Mozambique	1997	3 035	25.3	36.8	27.9	39.9	1.7
12. Tanzania	1999	1 588	18.4	38.7	20.1	42.9	3.0
13. Uganda	2000/01	3 282	9.9	36.2	27.3	37.2	1.6
14. Zambia	2001/02	3 475	30.2	44.9	38.4	47.7	1.5
15. Zimbabwe	1999	1 635	31.9	27.2	22.6	29.4	1.4
							
All 15 countries	NA^b^	41 341	21.5	36.1	27.2	38.5	1.7

^a^Children aged 1–35 months. Children with missing or inconsistent anthropometric measures are excluded.

^b^Not applicable

For a background, Table 2 also shows the percentage of sample children living in urban areas. The average proportion of urban children stands at 21.5%, with the highest value found in Côte d'Ivoire, Ghana, Nigeria, Zimbabwe and Zambia (30–33%), whereas the lowest proportion is recorded in Uganda, Burkina Faso, Malawi, Kenya and Tanzania (between 10 and 18%). Table 2 also displays the prevalence of malnutrition by place of residence. As can be noticed, more than 35% of the sample children are undernourished. This rate of stunting reaches almost 45–50% in Madagascar, Zambia and Malawi, and varies between 30% and 40% in the remaining countries with the exception of Togo, Côte d'Ivoire, Ghana and Zimbabwe, where it stands at 23–28%. Moreover, the prevalence of stunting is higher in rural areas compared to urban areas in all countries. Odds ratios (OR) of rural-urban differences in stunting vary from 1.5 or less in Madagascar, Zimbabwe, Zambia, Côte d'Ivoire, Chad and Kenya, to nearly 2.0 in Burkina Faso and Malawi, and even 3.0 in Tanzania, with average value (for the overall sample) of 1.7 (see Table 2).

### 3.2. Differences across urban and rural areas in socioeconomic inequalities

Table 3 shows the coefficients for multilevel models of socioeconomic inequalities in child malnutrition at the national level. The coefficients are in the expected direction and statistically significant in all countries (p < 0.10 in Madagascar; p < 0.01 in all other countries). This result which is in line with the rural to urban OR in Table 2, indicates that in all selected countries, children from poorer households are at substantially greater risk of malnutrition than their counterparts from wealthier households. The interaction of household wealth and area of residence is shown in Table 3. As can be seen, the coefficients are positive in all countries except Zambia, and to a lesser degree, Chad, indicating that disparities among socioeconomic groups are higher in urban areas than in rural settings. Further, the interaction term proves statistical significance in Mozambique, Madagascar, Uganda, Kenya, and Nigeria (p < 0.05) and Burkina Faso (p < 0.10). Derived coefficients and OR for urban and rural areas are shown in Cols. 3–6 of Table 3. Within-urban differentials in child malnutrition vary from 1.4 in Zambia to 3.8 in Mozambique, with a median value of 2.3 (in Malawi), whereas within-rural differentials range from 1.0 in Madagascar to 2.8 in Tanzania, with a median value of 1.7 in Cameroon.

**Table 3:** Coefficients and odds ratios for multilevel models of socioeconomic inequalities in child malnutrition by area of residence in 15 selected countries

			Within-urban inequities		Within-rural inequities	
				
	Inequities at the national level (coefficient) (1)	Interaction of SES and area of residence (coefficient) (2)	Coefficient (3)	Odds ratio (4)	Coefficient (5)	Odds ratio (6)
Central & Western Africa						
1. Burkina Faso	-0.346 ***	0.580 *	-0.824 ***	2.3	-0.244 *	1.3
2. Cameroon	-0.676 ***	0.458	-0.963 ***	2.6	-0.505 ***	1.7
3. Chad	-0.409 ***	-0.026	-0.399 **	1.5	-0.425 ***	1.5
4. Côte d'Ivoire	-0.754 ***	0.276	-0.884 ***	2.4	-0.608 *	1.8
5. Ghana	-0.454 ***	0.302	-0.655 **	1.9	-0.353 *	1.4
6. Nigeria	-0.741 ***	0.588 **	-1.117 ***	3.1	-0.529 ***	1.7
7. Togo	-0.675 ***	0.168	-0.809 ***	2.2	-0.641 ***	1.9
Eastern & Southern Africa						
8. Kenya	-0.732 ***	0.621 **	-1.219 ***	3.4	-0.598 ***	1.8
9. Madagascar	-0.204 *	0.722 **	-0.767 ***	2.2	-0.045	1.0
10. Malawi	-0.622 ***	0.288	-0.842 ***	2.3	-0.554 ***	1.7
11. Mozambique	-1.079 ***	0.734 **	-1.336 ***	3.8	-0.602 *	1.8
12. Tanzania	-1.066 ***	0.205	-1.248 ***	3.5	-1.043 ***	2.8
13. Uganda	-0.575 ***	0.664 **	-1.099 ***	3.0	-0.435 ***	1.5
14. Zambia	-0.442 ***	-0.164	-0.312	1.4	-0.476 ***	1.6
15. Zimbabwe	-0.507 ***	0.263	-0.716 **	2.0	-0.453 ***	1.6

**Note**: Coefficients of the uppermost category of household wealth or odds ratios between the uppermost and the lowermost categories of household wealth are used as a measure of socioeconomic inequalities.

*p < 0.10; **p < 0.05; ***p < 0.01.

Of interest in this study is the close examination of intra-urban inequities. Table 3 (Col. 4) indicates that the widest within-urban gaps (OR of 3.0 or higher) are to be found in Mozambique, Tanzania, Kenya, Nigeria and Uganda. At the other extreme, the narrowest gaps (around 2.0 or less) are recorded in Zambia, Chad, Ghana, and Zimbabwe. The associated coefficients are statistically significant in all countries except in Zambia.

### 3.3. What explains socioeconomic inequalities in urban areas?

The global view of urban inequities depicted in Cols 3–4 of Table 3, does not, however, take into account the complex set of individual, household and community characteristics which are linked to urban place of residence and may be, to a large extent, responsible for children's health status. Table 4 shows the change in intra-urban disparities in child malnutrition when different combinations of control variables are included in the models. Model 1 is the baseline model; Model 2 adds community SES to Model 1; Model 3 adds mother's and father's education to Model 1; Model 4 adds community SES and mother's and father's education to Model 1; Model 5 adds bio-demographic control variables to Model 4.

**Table 4:** Factors associated with intra-urban inequities in child malnutrition in 15 selected countries

	Intra-urban inequities				
	
	Model 1	Model 2	Model 3	Model 4	Model 5
Central & Western Africa					
1. Burkina Faso	-0.824 ***	-0.771 **	-0.466	-0.431	-0.597 *
2. Cameroon	-0.963 ***	-0.841 ***	-0.820 ***	-0.798 ***	-0.643 **
3. Chad	-0.399 **	-0.332 *	-0.216	-0.207	-0.447 **
4. Côte d'Ivoire	-0.884 ***	-0.620 **	-0.856 ***	-0.636 **	-0.707 **
5. Ghana	-0.655 **	-0.544	-0.560 *	-0.522	-0.605 *
6. Nigeria	-1.117 ***	-0.672 ***	-0.634 **	-0.356	-0.351
7. Togo	-0.809 ***	-0.624 **	-0.624 **	-0.502 *	-0.441
Eastern & Southern Africa					
8. Kenya	-1.219 ***	-1.125 ***	-0.936 ***	-0.883 ***	-0.951 ***
9. Madagascar	-0.767 ***	-0.912 ***	-0.555 **	-0.709 **	-0.823 **
10. Malawi	-0.842 ***	-0.780 ***	-0.644 ***	-0.615 ***	-0.721 ***
11. Mozambique	-1.336 ***	-1.227 ***	-1.185 ***	-1.007 **	-0.986 **
12. Tanzania	-1.248 ***	-1.204 ***	-1.061 ***	-1.052 ***	-0.808 **
13. Uganda	-1.099 ***	-0.937 ***	-0.994 ***	-0.874 ***	-0.888 ***
14. Zambia	-0.312	-0.175	-0.210	-0.111	0.013
15. Zimbabwe	-0.716 **	-0.715 **	-0.622 *	-0.647 *	-0.764 **

**Note**: Coefficients of the uppermost category of household wealth are used as a measure of socioeconomic inequalities.

Model **1 **is the baseline model; Model **2 **adds community SES to Model 1; Model **3 **adds mother's and father's education to Model 1; Model **4 **adds community SES and mother's and father's education to Model 1; Model **5 **adds bio-demographic control variables to Model 4.

*p < 0.10; **p < 0.05; ***p < 0.01.

Our results show that controlling for community SES (Model 2) resulted in loss of statistical significance of within-urban differentials in child malnutrition in only one country (Chad). Adjusting for maternal and father education (Model 3) led to loss of statistical significance in two countries (Burkina Faso and Chad), and controlling for all three measures of SES (Model 4) produced loss of statistical significance of the intra-urban gaps in child health in four countries (Burkina Faso, Chad, Ghana and Nigeria). Surprisingly, controlling for the mother-, and child-level covariates (Model 5) resulted in increased within-urban differentials in Burkina Faso and Chad to statistical significance at the level of 0.10. Overall, within-urban differentials in child malnutrition were almost explained by our measured covariates in only two countries (Nigeria and Togo).

## 4. Discussion

This study has examined and documented differences across urban and rural areas in child health inequities. The first objective of the paper was to compare the scale of socioeconomic inequalities in child malnutrition across urban and rural areas. Our results show that in all countries and areas (urban or rural), children from the poorest households stand greater risk to be undernourished, than their counterparts in the most privileged households. Most studies that have used socioeconomic index [21,22,25] or socioeconomic factors [16,18,23] have reported similar results. More importantly, this study shows that while malnutrition is, on average, higher in rural compared to urban areas -a finding reported by other authors [7,43]- socioeconomic inequalities are, to a large extent, higher in cities than in rural areas. Many studies on socioeconomic inequalities in health have also shown evidence of higher heterogeneity of urban areas compared to rural settings, with the former harboring pockets of severe poverty and deprivation, and exhibiting substantial concentrations of ill-health among the poor [5,6,9,21].

Linking intra-urban disparities in Col. 4 of Table 3 to urban malnutrition in Table 2 shows that some countries like Mozambique, Nigeria and Uganda exhibit higher urban malnutrition rates and higher urban socioeconomic inequalities, whereas others like Ghana, Zimbabwe, Togo and Burkina Faso record lower values in both counts. Between these two extremes, Zambia, Chad, Madagascar, Tanzania, Côte d'Ivoire and Cameroon have lower values in one dimension and higher levels in the other. Results in Tanzania and Mozambique are worthy of attention. Despite its fastest urban population growth, Tanzania has a relatively low level of urban malnutrition, the largest urban-rural gap in malnutrition (see rural to urban odds ratio in Table 2), and a modest level of intra-urban inequalities in malnutrition. Like Tanzania, Mozambique witnessed faster urban population growth, coupled with increased per capita GDP. Yet, it has higher urban malnutrition, and more importantly, it records the largest intra-urban differences in child undernutrition. This finding indicates that the magnitude of within-urban inequities in child health is not merely a result of urban population growth, and suggests that well-designed policies can reduce these inequities even in countries facing urban explosion.

Another issue examined in this paper has been the magnitude of within-urban inequalities in child malnutrition across countries. Our results show large but varying levels of inequalities across countries, which are even larger than urban-rural differentials in malnutrition. Comparing within-urban differentials in child malnutrition to rural-urban differentials in malnutrition shown in Table 2 reveals that within-urban differentials are of higher magnitude compared to urban-rural differentials in all countries except Chad and Zambia, the only countries where the within-urban gap in stunting is not larger than the within-rural one. Indeed, rural to urban OR in the prevalence of child stunting vary from 1.2 in Madagascar to 3.0 in Tanzania with a median value of 1.6 in Uganda, whereas within-urban differentials in malnutrition range from 1.4 (Zambia) to 3.8 (Mozambique), for a median value of 2.3 (Burkina Faso), as indicated earlier.

This finding is in line with work of Menon *et al*. [5], which showed that intra-urban differentials in child stunting were larger than overall urban-rural differences in 8 out of 11 developing countries from SSA, Asia and Latin America. The fact that within-urban gaps in child health are larger than within-rural gaps, and even than overall urban-rural gaps, suggests that using global urban-rural prevalence to characterize child malnutrition may be misleading, since urban average could mask large differentials among socioeconomic groups in urban areas. These conclusions are in accordance with those of a number of studies which have demonstrated the existence of substantial concentrations of ill-health among the urban poor [5,9,21]. They suggest that policies and programs geared at improving children's welfare should specifically include targeting the urban poor.

The third issue investigated in this work has been the extent to which within-urban differentials are explained by the characteristics of communities, households and individuals. Our data show that the influences of mother's and father's education, community SES, and bio-demographic variables are relatively modest in explaining inequities in child stunting among urban dwellers. This result corroborates findings from other studies which have demonstrated that household income is a key and independent determinant of food insecurity and malnutrition [22,44,45]. The fact that adjusting for bio-demographic covariates produced an increase of urban inequities in most countries is quite surprising. Similar findings have been reported in other developing countries like Brazil where Sastry found that important differences in child mortality by place of residence were revealed by controlling for community characteristics [36].

### Limitations of the study

One of the problems in cross-country studies on urban/rural differentials is the classification of localities as urban or rural. Some countries classify in terms of administrative boundaries, others in terms of agglomerations. Other criteria used include population size, population density, or a combination of several of these criteria [46]. Though this variety of urban/rural classifications undoubtedly weakens any cross-country comparisons, a uniform definition cannot capture the large variety of urban and rural situations across countries with such wide disparities of economic and social development as those used in this study. A second limitation of this analysis relates to our constructed community SES. Though the variable is worthy of interest given the growing body of research on the effects of neighborhood characteristics on health [22,37,38], it should be noted that other community correlates likely to affect child health were not included in the analysis. These include variables that were not measured or not measurable such as food availability, agricultural and climate characteristics, air pollution, and epidemiologic data. The fact that community-level variance demonstrates statistical significance in all countries except Burkina Faso and Zimbabwe (not shown) is supportive of the possible effect of unobserved community factors.

## 5. Conclusion

This study has used standardized measures of SES defined at the household and community levels to document the scale of inequities in child malnutrition in SSA. It has shown that across countries in SSA, though socioeconomic inequalities in stunting do exist in both urban and rural areas, they are significantly larger in urban areas. Our results further show that intra-urban differences in child malnutrition are larger than overall urban-rural differentials in child malnutrition, and that they vary across countries, even among those with comparable levels of development. Finally, our results indicate that maternal and father's education, community SES and other measurable covariates at the mother and child levels only explain a slight part of the within-urban differences in child malnutrition.

Overall, the results of this piece of work suggest that specific policies geared at preferentially improving the health and nutrition of the urban poor should be implemented, so that while targeting the best attainable average level of health, reducing gaps between population groups is also on target [14]. Haddad *et al*. note that intra-urban differentials in health are not sufficiently highlighted [6], and as Garrett & Ruel purposely point out, most programs to alleviate food insecurity and malnutrition are designed for rural areas, despite increasing evidence of declining living conditions in most cities of SSA [44]. To successfully monitor the gaps between urban poor and non-poor, existing data collection programs, such as the DHS and other nationally representative surveys, should be re-designed to capture the changing patterns of the spatial distribution of population. Indeed, these programs usually exclude the slum areas since they are considered illegal settlements, and when they are included, the sample size is often too small to allow any reasonable slum specific estimates.

## Declaration of competing interests

The author(s) declare that they have no competing interests.

## Notes

^1^In this paper the terms "*socioeconomic inequalities*" and "*inequities*" are used interchangeably. We do share the view that *health inequality *is a generic term used to designate differences and disparities in the health achievements of individuals and groups, whereas the term *health inequities *refers to inequalities that are unjust or unfair.

^2^HDI is a composite index based on three dimensions: health (longevity), education (literacy rate), and resource (standard of living). Countries are ranked in decreasing order of human development index (e.g. rank 1 corresponds to the highest human development level).
